# Air-Assisted Descemet Stripping Automated Endothelial Keratoplasty with Posterior Chamber Fixation of an Aphakic Iris-Claw Lens

**Published:** 2010-07

**Authors:** Farid Karimian, Mohammad-Mehdi Sadoughi

**Affiliations:** Ophthalmic Research Center, Labbafinejad Medical Center, Shahid Beheshti University of Medical Sciences, Tehran, Iran

**Keywords:** Descemet Stripping Endothelial Keratoplasty, Lens Implantation, Intraocular

## Abstract

Endothelial keratoplasty (EK) is the most exciting recent development in corneal transplantation. It has experienced surprisingly rapid growth in a very short period of time. One of the indications for EK is pseudophakic bullous keratopathy. However, concomitant intraocular lens (IOL) exchange, if indicated, may prove challenging. Some surgeons routinely perform IOL exchange with a scleral-fixated posterior chamber IOL, together with Descemet’s stripping endothelial keratoplasty (DSEK); however, this combined procedure is time-consuming, difficult and fraught with complications. Another option is aphakic Artisan IOL fixation, but this is usually not acceptable because of the increased risk of endothelial cell loss and difficulty in filling the anterior chamber with the air bubble. Herein, we introduce a new technique for IOL exchange with an aphakic Artisan IOL fixated posterior to the iris, combined with DSEK. This surgical technique was designed to preserve anterior segment anatomic features as much as possible.

## INTRODUCTION

Descemet stripping automated endothelial keratoplasty (DSAEK) has become a popular alternative to penetrating keratoplasty in recent years. Advantages of DSAEK include faster visual recovery, minimal change in astigmatism, lower risk of wound dehiscence, and a tectonically stable globe. Furthermore, the risk of intraoperative expulsive suprachoroidal hemorrhage is minimized. Its main disadvantages remain to be a high rate of graft dislocation and an increased rate of endothelial cell loss.

In 1998, Melles presented his novel technique in which corneal transplantation was performed for replacement of diseased endothelium. This new technique, which he named posterior lamellar keratoplasty (PLK), involved dissecting a posterior recipient lamella and transplanting an unsutured posterior lamellar donor disc consisting of posterior stroma, Descemet’s membrane, and endothelium through a scleral incision.^1^

In 2000, Terry performed the first endothelial keratoplasty in the United States and called it deep lamellar endothelial keratoplasty (DLEK).^2^

In 2003, Melles reported how a folded donor transplant insertion could be combined with removing the recipient endothelial layer by means of stripping Descemet’s membrane.^3^ This obviated the need for stromal dissection of the recipient which had been the most challenging aspect of the procedure.

In 2005, Price refined methods to score Descemet’s membrane and improve donor adherence, the so-called Descemet’s stripping endothelial keratoplasty (DSEK), and expanded the indications to include treatment of failed grafts due to endothelial decompensation.^4^–^6^

In 2006, Gorovoy reported the use of a microkeratome instead of manual dissection of the donor graft and called the procedure Descemet’s stripping automated endothelial keratoplasty (DSAEK).^7^

Indications for DSAEK include: (1) Fuchs’ endothelial dystrophy, (2) pseudophakic bullous keratopathy, (3) aphakic bullous keratopathy, (4) failed penetrating keratoplasty, and (5) iridocorneal endothelial (ICE) syndrome. Contraindications to DSAEK are visually significant stromal scarring or opacities in the anterior cornea.

Graft detachment is the most frequent early postoperative complication following DSAEK (10–35%), particularly for novice surgeons. Graft detachment is usually detectable during the first day or two after surgery, but may rarely occur up to several weeks later.

Meisler et al^8^ used an air-fluid exchange system to promote graft adhesion during DSAEK. In their report, they used a 30-gauge needle fixed to the recipient limbus to introduce air into the anterior chamber during the procedure. Similarly, Mehta et al^9^ used an anterior chamber (AC) maintainer at the recipient limbus, attached to a 3-way tap connected to an air syringe to keep the AC air-filled during descemetorhexis.

The surgical correction of aphakic eyes with corneal edema without adequate capsular support is controversial. A debate persists between the choice of an angle-supported anterior chamber intraocular lens or a sutured posterior chamber intraocular lens (IOL), or recently, iris-claw IOLs (e.g., Artisan).^10^–^12^ Scleral-fixated IOLs incur certain disadvantages, including difficult technique, longer surgical time and excessive intraocular manipulation. Angle-supported anterior chamber IOLs are also associated with complications, such as bullous keratopathy due to presence of haptics in the iridocorneal angle and continous endothelial cell loss.^13^

In 1986, the first iris-claw IOL was implanted in a phakic eye by Worst and Fechner^14^. Some studies have indicated favorable visual outcomes and a low incidence of intraoperative and postoperative complications with these IOLs.^15^–^17^ The Artisan Aphakia IOL, (Ophtec BV, Groningen, the Netherlands), is one of the latest versions of this type of iris-fixated IOLs. It is a single-piece polymethylmethacrylate (PMMA) lens which is attached to the iris with clip haptics on both sides of the optic. The haptics are enclavated to the mid-periphery of the iris.^18^ Implantation of the iris-claw IOL behind the iris will better preserve the anatomy of the anterior segment and may result in less endothelial cell loss.

Some surgeons use iris-sutured posterior chamber IOLs during keratoplasty in cases for which access and visualization are good; however, this procedure is significantly difficult when visibility is limited and access is restricted, as in cases of DSAEK. Some surgeons opt to leave the offending anterior chamber IOL in place during DSAEK in eyes with bullous keratopathy due to technical reasons.^19^

Lake and Rostron^20^ attempted to overcome these problems by anterior chamber fixation of an Artisan during DSAEK by their modified technique. Because Artisan Aphakia IOLs are placed in the anterior chamber, long-term observation is required to ensure that the donor endothelium is not affected when implantation is combined with DSAEK. The disadvantage of PMMA IOLs in the anterior chamber, other than the increased risk of graft rejection, is decreased anterior chamber volume, which makes graft unfolding more difficult^19^,^20^, especially when the endothelial graft touches the IOL margin.

In this article, we report a new combination of surgical techniques for air- assisted DSAEK and posterior chamber Artisan Aphakia IOL implantation/exchange in patients with aphakic/pseudophakic bullous keratopathy. To the best of our knowledge, there is no report on such a technique in the literature

## SURGICAL TECHNIQUE

The designated eye is prepared and draped in a sterile manner. A one-millimeter tangential inferior paracentesis is performed at 6 o’clock entering the anterior chamber in the posterior limbal area parallel to the iris. Then, a 5.5 mm mid-limbal incision is created at 12 o’clock and two vertical paracenteses at 10 and 2 o’clock positions are performed for entrance of the enclavation needle. If there is an angle supported IOL, it is removed through the main incision. The recipient epithelium can be removed to improve visualization. Bi-manual anterior vitrectomy is performed before iris-claw IOL insertion to clear the anterior chamber and retropupillary area from any vitreous.

**Figure 1:**
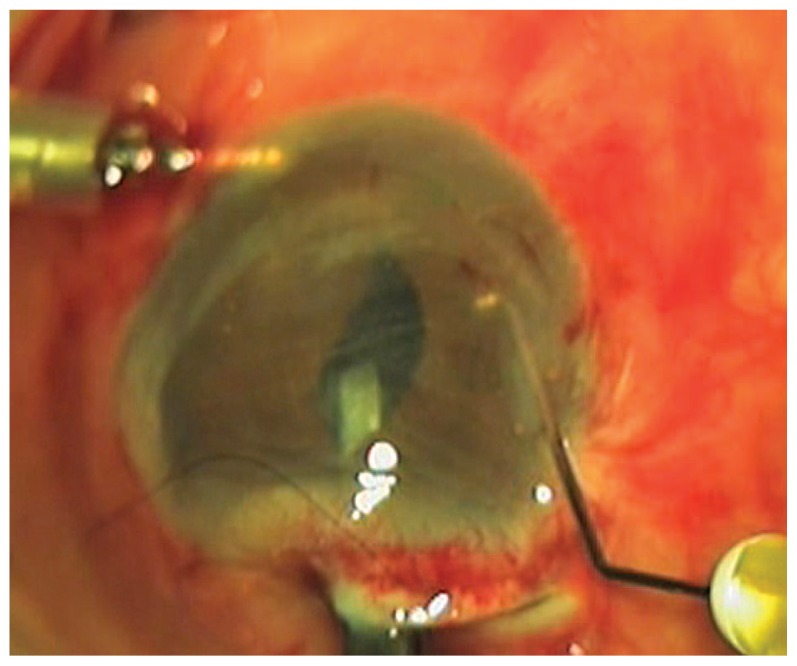
Fixation of the aphakic Artisan lens with forceps and enclavation while the haptic is indenting the posterior surface of the iris.

Viscoelastic material (1% sodium hyaluronate) is injected behind the pupillary plane to tamponade vitreous remnants. The iris-claw Artisan Aphakia IOL is inserted through the incision upside-down, in a reverse position. A lens holding forceps is introduced through the incision and the Artisan is grasped. One half of the IOL is slipped through the pupil with the haptics positioned at 3 and 9 o’clock and maintained horizontally using the forceps. By gentle forward elevation of the Artisan, the imprint of its haptic and site of enclavation will be made visible through the iris. At the same time, through the paracentesis and using an enclavation needle, the iris is enclavated into the IOL haptic (Fig. 1). While the IOL is still firmly grasped with forceps, the other half of the IOL is slipped under the iris and the same maneuver is repeated on the other side, achieving perfect IOL centration under the pupil. Afterwards, a peripheral iridectomy is performed. Up to this stage, the AC can be filled with BSS through the inflow.

After IOL implantation/exchange, DSAEK is performed. The donor cornea is prepared first, followed by surgery on the recipient. Dissection of the lamellar donor disc using an artificial anterior chamber may be performed at the eye bank (precut tissue) or by the surgeon using a microkeratome. Complete flap resection is performed to leave an 8.5 to 9.0 mm stromal bed. The donor tissue is transferred to a Barron punching system (Katena, Denville, USA) and punched 8.0 to 9.0 mm in diameter according to the recipient’s corneal diameter and area of stripped Descemet’s membrane.

**Figure 2:**
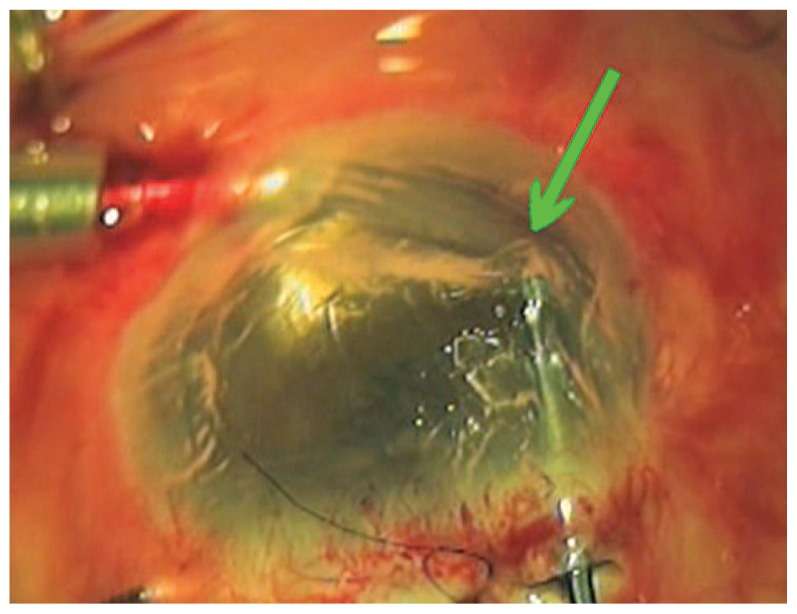
Good visualization of Descemet’s membrane during stripping when the AC is formed with an air bubble.

In the next step, the corneal surface can be marked lightly with a 9-mm marker to outline where to strip recipient Descemet’s membrane and highlight the fixation area of the donor tissue. The air inflow is attached to the air-fluid exchange machine (Accurus, Alcon Labs, Fort Worth, USA) set at 40 mmHg of pressure while monitoring intraocular pressure and anterior chamber depth. Descemet’s membrane is stripped in a circular pattern (descemetorhexis) under the area of the epithelial reference mark with an anteriorly bent needle or custom-made instrument (modified Price–Sinskey hook). Descemet’s membrane and endothelium is stripped completely from the marked area and removed from the AC using a 45° or 90° Descemet’s strip instrument. By creating good contrast, air provides adequate visualization of Descemet’s membrane during stripping and there is no need for trypan blue staining (Fig. 2).

**Figure 3:**
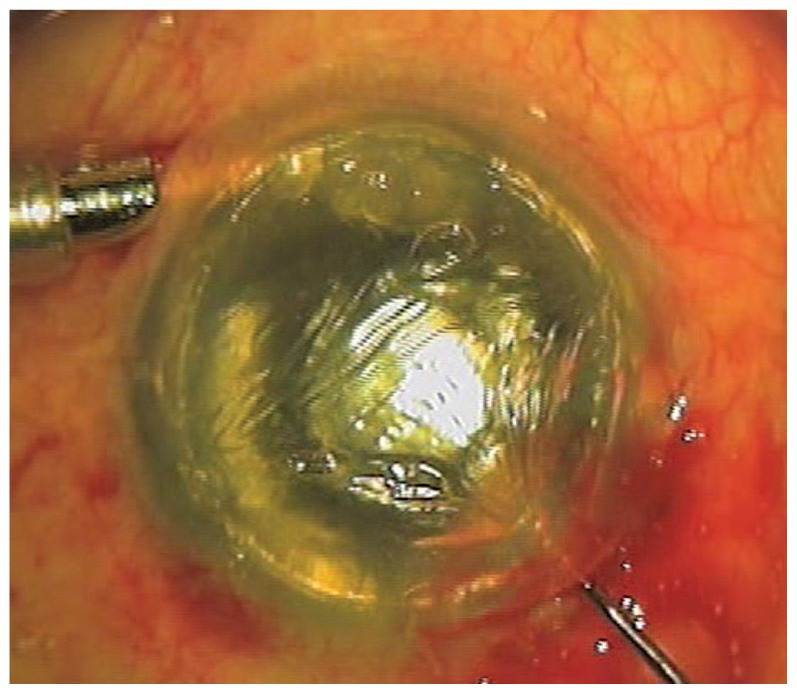
Stretching the edges of the donor lenticule eliminates tissue wrinkling.

The trephined donor corneal lenticule (containing posterior stroma, Descemet’s membrane, and endothelium) is brought into the operative field, a small amount of viscoelastic is placed on the endothelial surface, and the disc is folded over itself into an asymmetric 60:40 configuration (the so-called “taco” shape) with the endothelial side inward. Using long DSAEK forceps, the donor disc is gently grasped and inserted into the eye. During insertion, air inflow pressure is reduced to allow the disc to remain in the anterior chamber. On entrance, the disc adheres to the posterior stroma while still folded, and air flow from beneath enhances adhesion. Excessively high air pressure will lead to extrusion of the donor disc from the anterior chamber. The incision is closed with 10-0 nylon sutures, and the anterior chamber is maintained by continuous inflow of air. A modified Price-Sinskey hook is used to complete unfolding, achieve donor centration, and stretch the donor tissue at its edges to eliminate tissue wrinkles (Fig. 3).

While the AC is still pressurized with air, the corneal surface is massaged to remove any fluid from the donor/recipient interface. Air is left in place for 10 minutes. To prevent pupillary block, 20 to 30% of the anterior chamber volume is replaced by BSS. Next the air inflow is removed and the paracentesis is closed with a single 10-0 nylon suture. The patient should remain in supine position for 30 to 60 minutes in the recovery room to allow the remaining air bubble to push the donor tissue up against the recipient cornea. Postoperatively, a paracentesis release can be applied to prevent pupillary block by ensuring that the remaining air bubble is above the pupillary border in sitting position.

## DISCUSSION

For decades, anterior chamber angle supported IOLs^21^,^22^ and scleral or iris-fixated posterior chamber IOLs^23^,^24^ have been the most popular types of lenses used for secondary IOL implantation in the absence of capsule support. The first report of retropupillary fixation of an iris-claw IOL in aphakia was published by Rijneveld et al.^25^ Later, Mohr et al^26^ published the second report on retropupillary iris-claw IOL fixation in 48 aphakic patients. No major complication was observed and the new technique was shown to be superior (in terms of simplicity, reliability, and anatomical results) to other techniques. Another study also confirmed the relative safety of posterior iris fixation of the iris-claw IOL through a scleral tunnel incision in patients without adequate capsular support.^27^

Herein we report DSAEK in eyes with bullous keratopathy combined with posterior chamber iris fixation of an Artisan Aphakia iris-claw lens performed as a secondary IOL implantation procedure in aphakic eyes, or for exchange of anterior chamber angle-supported lenses in pseudophakic eyes. This surgical technique was designed to restore anterior segment anatomy as much as possible, regarding the fact that the ideal position for an IOL is posterior to the iris plane. The rationale behind this technique is to maximize the distance between the IOL and the donor endothelium while avoiding anterior chamber angle structures. Some authors have expressed concern about the potential of the iris-claw lens to damage the iris or the corneal endothelium.^28^,^29^ Furthermore, if enclavation fails, dislocation of the iris-claw IOL into the vitreous cavity may occur. Such a complication may occur during the procedure in case of inadequate holding of the IOL with forceps. Placing only half of the IOL behind the iris during sequential enclavation ensures proper IOL positioning and prevents its dislocation. However, inadequate enclavation of iris tissue may cause slippage of the iris-claw haptics, especially in the long-term.

The use of air during DSAEK has been previously reported. In the report by Mehta et al^9^, air was used in the anterior chamber using a syringe only during descemetorhexis. In another report, Meisler et al^8^ used air inflow through a 30-gauge needle. Our technique uses continuous air inflow supplied by an air-fluid exchange system during all stages of DSAEK including descemetorhexis, donor disc insertion, and enhancement of adhesion. We performed ten cases of such combined procedures with about 6 months of follow up. Posterior chamber fixated iris-claw Artisan Aphakia lenses have remained attached and there has been no case of IOL dislocation. In addition, these eyes have maintained clear corneas and successfully attached donor lenticules, no case of graft dislocation has occurred and only few eyes have required rebubbling. Maintenance of air in the anterior chamber during the procedure seems to dry the donor-recipient interface, hence enhancing adhesiveness of both surfaces. One caveat is that intraoperative delay in proper positioning of the donor lenticule may complicate manipulation which is due to immediate formation of strong adhesions.

In summary, DSAEK combined with posterior chamber iris fixation of the Artisan Aphakia IOL appears to be a safe method for management of aphakic/pseudophakic corneal edema. Prospective studies on a larger number of cases are warranted to determine the long-term visual outcomes and possible complications.

## References

[1] Melles GR, Eggink FA, Lander F, Pels E, Rietveld FJ, Beekhuis WH (1998). A surgical technique for posterior lamellar keratoplasty. Cornea.

[2] Terry MA, Ousley PJ (2001). Deep lamellar endothelial keratoplasty in the first United States patients: early clinical results. Cornea.

[3] Melles GR, Wijdh RH, Nieuwendaal CP (2004). A technique to excise the descemet membrane from a recipient cornea (descemetorhexis). Cornea.

[4] Price FW, Price MO (2005). Descemet’s stripping with endothelial keratoplasty in 50 eyes: a refractive neutral corneal transplant. J Refract Surg.

[5] Price FW, Price MO (2006). Descemet’s stripping with endothelial keratoplasty in 200 eyes: Early challenges and techniques to enhance donor adherence. J Cataract Refract Surg.

[6] Price FW, Price MO (2006). Endothelial keratoplasty to restore clarity to a failed penetrating graft. Cornea.

[7] Gorovoy MS (2006). Descemet-stripping automated endothelial keratoplasty. Cornea.

[8] Meisler DM, Dupps WJ, Covert DJ, Koenig SB (2007). Use of an air-fluid exchange system to promote graft adhesion during Descemet’s stripping automated endothelial keratoplasty. J Cataract Refract Surg.

[9] Mehta JS, Hantera MM, Tan DT (2008). Modified air-assisted descemetorhexis for Descemet-stripping automated endothelial keratoplasty. J Cataract Refract Surg.

[10] Evereklioglu C, Er H, Bekir NA, Borazan M, Zorlu F (2003). Comparison of secondary implantation of flexible open-loop anterior chamber and scleral-fixated posterior chamber intraocular lenses. J Cataract Refract Surg.

[11] Ozmen AT, Dogru M, Erturk H, Ozcetin H (2002). Transsclerally fixated intraocular lenses in children. Ophthalmic Surg Lasers.

[12] Zetterström C, Lundvall A, Weeber H, Jeeves M (1999). Sulcus fixation without capsular support in children. J Cataract Refract Surg.

[13] Sawada T, Kimura W, Kimura T, Suga H, Ohte A, Yamanishi S (1998). Long-term follow-up of primary anterior chamber intraocular lens implantation. J Cataract Refract Surg.

[14] Fechner PU, Worst JGF (1989). A new concave intraocular lens for the correction of high myopia. Eur J Implant Ref Surg.

[15] Fechner PU, van der Heijde GL, Worst JG (1989). The correction of myopia by lens implantation into phakic eyes. Am J Ophthalmol.

[16] Menezo JL, Martinez MC, Cisneros AL (1996). Iris-fixated Worst claw versus sulcus-fixated posterior chamber lenses in the absence of capsular support. J Cataract Refract Surg.

[17] Güell JL, Velasco F, Malecaze F, Vázquez M, Gris O, Manero F (2005). Secondary Artisan-Verysise aphakic lens implantation. J Cataract Refract Surg.

[18] Lovisolo CF, Reinstein DZ (2005). Phakic intraocular lenses. Surv Ophthalmol.

[19] Groat B, Ying MS, Vroman DT, Fernandez de Castro LE (2007). Descemet-stripping automated endothelial keratoplasty technique in patients with anterior chamber intraocular lenses [video report]. Br J Ophthalmol.

[20] Lake DB, Rostron CK (2008). Management of angle-supported intraocular lens and iridectomy in Descemet-stripping endothelial keratoplasty. Cornea.

[21] Weene LE (1993). Flexible open-loop anterior chamber intraocular lens implants. Ophthalmology.

[22] Rattigan SM, Ellerton CR, Chitkara DK, Smerdon DL (1996). Flexible open-loop anterior chamber intraocular lens implantation after posterior capsule complications in extracapsular cataract extraction. J Cataract Refract Surg.

[23] McCluskey P, Harrisberg B (1994). Long-term results using scleral-fixated posterior chamber intraocular lenses. J Cataract Refract Surg.

[24] Trimarchi F, Stringa M, Vellani G, Iato MS (1997). Scleral fixation of an intraocular lens in the absence of capsular support. J Cataract Refract Surg.

[25] Rijneveld WJ, Beekhuis WH, Hassman EF, Dellaert MM, Geerards AJ (1994). Iris claw lens: anterior and posterior iris surface fixation in the absence of capsular support during penetrating keratoplasty. J Refract Corneal Surg.

[26] Mohr A, Hengerer F, Eckardt C (2002). Retropupillary fixation of the iris claw lens in aphakia. 1 year outcome of a new implantation technique. Ophthalmologe.

[27] Baykara M, Ozcetin H, Yilmaz S, Timuçin OB (2007). Posterior iris fixation of the iris-claw intraocular lens implantation through a scleral tunnel incision. Am J Ophthalmol.

[28] Menezo JL, Cisneros AL, Rodriguez-Salvador V (1998). Endothelial study of iris-claw phakic lens: four year follow-up. J Cataract Refract Surg.

[29] Menezo JL, Aviño JA, Cisneros A, Rodriguez-Salvador V, Martinez-Costa R (1997). Iris-claw phakic intraocular lens for high myopia. J Refract Surg.

